# *Graptopetalum paraguayense* Extract Ameliorates Proteotoxicity in Aging and Age-Related Diseases in Model Systems

**DOI:** 10.3390/nu13124317

**Published:** 2021-11-29

**Authors:** Yan-Xi Chen, Phuong Thu Nguyen Le, Tsai-Teng Tzeng, Thu-Ha Tran, Anh Thuc Nguyen, Irene Han-Juo Cheng, Chi-Ying F. Huang, Young-Ji Shiao, Tsui-Ting Ching

**Affiliations:** 1 Institute of Biopharmaceutical Sciences, National Yang Ming Chiao Tung University, Taipei 112, Taiwan; sunkist210@hotmail.com (Y.-X.C.); fly23242530@hotmail.com (T.-T.T.); anhthucnguyen1004@gmail.com (A.T.N.); 2 Taiwan International Graduate Program in Molecular Medicine, National Yang Ming Chiao Tung University and Academia Sinica, Taipei 115, Taiwan; thuphuonglenguyen96@gmail.com (P.T.N.L.); tranthuha164@gmail.com (T.-H.T.); 3 Institute of Brain Science, National Yang Ming Chiao Tung University, Taipei 112, Taiwan; ihjcheng@gmail.com; 4 National Research Institute of Chinese Medicine, Ministry of Health and Welfare, Taipei 112, Taiwan

**Keywords:** GP extract, neurodegenerative disease, Alzheimer’s disease, amyloid-β, autophagy, longevity

## Abstract

Declines in physiological functions are the predominant risk factors for age-related diseases, such as cancers and neurodegenerative diseases. Therefore, delaying the aging process is believed to be beneficial in preventing the onset of age-related diseases. Previous studies have demonstrated that *Graptopetalum paraguayense* (GP) extract inhibits liver cancer cell growth and reduces the pathological phenotypes of Alzheimer’s disease (AD) in patient IPS-derived neurons. Here, we show that GP extract suppresses β-amyloid pathology in SH-SYS5Y-APP_695_ cells and APP/PS1 mice. Moreover, AMP-activated protein kinase (AMPK) activity is enhanced by GP extract in U87 cells and APP/PS1 mice. Intriguingly, GP extract enhances autophagy in SH-SYS5Y-APP_695_ cells, U87 cells, and the nematode *Caenorhabditis elegans*, suggesting a conserved molecular mechanism by which GP extract might regulate autophagy. In agreement with its role as an autophagy activator, GP extract markedly diminishes mobility decline in polyglutamine Q35 mutants and aged wild-type N2 animals in *C. elegans*. Furthermore, GP extract significantly extends lifespan in *C. elegans*.

## 1. Introduction

Aging is a normal physical process characterized by a general decline in physiological functions and behavioral capacity, leading to reduced vitality and eventually death [1,2]. As humans age, cellular damages accumulate, increasing the risk of disease formation. Among these age-related diseases, neurodegenerative diseases, such as Alzheimer’s disease (AD), Parkinson’s disease, and Huntington’s disease (HD), have garnered much attention due to the lack of effective treatment and accompanied economic burdens.

AD is the most common cause of dementia in people who are older. Amyloid plaques and neurofibrillary tangles (NFTs) in the brain, composed of abnormally folded amyloid-β42 (Aβ42) and phosphorylated tau proteins, are the pathological hallmarks of AD [3]. Autophagy has been recognized as a critical cellular mechanism in maintaining cellular homeostasis by degrading aggregated proteins and damaged organelles [4]. Recently, autophagy has been shown to mediate Aβ metabolism and tau assembly [5]. Numerous studies also demonstrate that autophagy dysfunction has been indicated in AD progression [6,7,8,9]. Furthermore, several pieces of evidence suggest that enhancing autophagy could promote the degradation of pathologic protein aggregates in AD and HD models [10,11,12,13,14].

Loss of protein homeostasis is a key hallmark of aging [2]. Thus, autophagy activation is also suggested to be beneficial for lifespan or health span in animals. Consequently, most of the interventions that extend lifespan in model organisms usually elevate autophagic activity [15]. Several lines of evidence have demonstrated that autophagy machinery is required for longevity regulation in animal models [16]. Recently, studies have shown that genetic activation of autophagy prolongs lifespan in mice [17,18], indicating the plausible application of autophagy activators in delaying the aging process and the onsets of age-related diseases.

*Graptopetalum paraguayense* (GP) is an edible succulent plant. In Taiwan, GP has been used as a medical herb to prevent liver disorders and lower blood pressure. Recent research has revealed that an extract of GP, HH-F3, could inhibit the proliferation of liver cancer cells, could lessen liver fibrosis in rats, and could reduce the secretion of Aβ and the phosphorylation of Tau proteins in induced pluripotent stem cell (iPSC)-derived neurons from AD patients [19,20,21,22]. This study further investigated the underlying mechanisms by which GP extracts reduce AD-associated pathological phenotypes in neuroblastoma SH-SYS5Y-APP_695_ cells and APP/PS1 mice. Furthermore, we demonstrated that GP extracts could reduce the mobility decline and could extend lifespan in *C. elegans*.

## 2. Materials and Methods

### 2.1. Preparation of GP Extract

The extraction method of GP HH-F3 was established previously [22,23]. In brief, frozen GP leaves were ground and lyophilized at −20 °C. Next, 15 g of lyophilized GP powder was mixed with 100 mL 100% ethanol for 5 min and then centrifuged at 1500× *g* for 5 min. The pellet was suspended in 10 mL of 30% dimethyl sulfoxide (DMSO), followed by 9300× *g* centrifuge for 5 min. The supernatant was fractionated into four fractions (F1–F4) by a Sephadex LH-20 column. The F3 fraction, termed HH-F3, was identified to be the active fraction.

### 2.2. Cell Culture

Human neuroblastoma SH-SY5Y cells were maintained in MEM/F12 (Gibco BRL, Grand Island, NY, USA) with 10% fetal bovine serum (FBS, Gibco BRL), 100 U/mL penicillin, and 10 μg/mL streptomycin sulfate. SH-SY5Y cells were stably transfected with the vector containing the full-length APP_695_ isoform. Stable clones with plasmid expression were maintained by growing cells in the selective medium containing G418. Human GBM cell line U87 was maintained in Dulbecco’s Modified Eagle Medium (Gibco BRL), supplemented with 10% FBS and 1% penicillin-streptomycin (Gibco BRL). Human colorectal cancer cell line HT-29 was cultured in McCoy’s 5A medium (Gibco BRL) supplemented with 10% FBS and 1% penicillin-streptomycin. All cells were maintained at 37 °C in 5% CO_2_. Cells were seeded at the density of 3 × 10^5^ cells/6 cm dish for at least 16 h before drug treatment.

### 2.3. Cell Viability Assay

The colorimetric MTT metabolic activity assay was used to determine cell viability.

Cells were incubated with minimum essential medium containing 0.5 mg/mL MTT (Sigma-Aldrich, St. Louis, MO, USA) for one hour. After incubation, the medium was aspirated, and the resultant formazan crystals were dissolved in DMSO. The absorbance intensity at 600 nm was measured by a microplate reader.

### 2.4. Extracellular Aβ1-40 and Aβ1-42 Detection

The SH-SY5Y-APP_695_ cells were treated with 0, 10, 30, and 50 μg/mL HH-F3 for 24 h. The conditioned medium was then harvested and centrifuged in the presence of 1 mM phenymethylsulfonyl fluoride (PMSF). Aβ1-40 and Aβ1-42 accumulation was assayed by Amyloid beta 40 human ELISA kit (KHB3481, Invitrogen, Waltham, MA, USA) and Amyloid beta 42 human ELISA kit (KHB3442, Invitrogen), respectively.

### 2.5. Western Blot Assay

The cells were lysed in buffer containing 50 mM HEPES, 2.5 mM EDTA, 1 mM PMSF, 5 μg/mL aprotinin, and 10 μg/mL leupeptin; 30 μg of protein lysate were electrophoresed on 8 or 10% SDS-PAGE gels and transferred to methanol-activated PVDF membranes. The membranes were blocked with 5% non-fat skim milk and incubated with primary antibodies at 4 °C overnight. Western blotting was visualized by peroxidase-conjugated secondary antibodies and ECL chemiluminescent substrate (Immobilon Western Chemiluminescent Substrate, Millipore, Burlington, MA, USA). The quantification of target protein bands with reference to control bands (for each concentration) used the ImageJ Gel Analysis program.

The following primary antibodies were used: anti-Aβ1-40 (A-8326, Sigma-Aldrich), anti-Aβ1-16 (MAB 5208. Merck, Kenilworth, NJ, USA), anti-APP-CTF (AB5352, Merck), anti-IDE (AB9210, Merck), anti-NEP (AB5468, Merck), anti-LC3 (2775, Cell Signaling Technology, Danvers, MA, USA), anti-p62 (8025S, Cell Signaling Technology), anti-p-AMPK(Thr172) (2535S, Cell Signaling Technology), anti-AMPK (2532S, Cell Signaling Technology), anti-GAPDH (GTX100118, GeneTex, Hsinchu, Taiwan), and anti-actin (AC-15, Novus, Centennial, CO, USA). Secondary antibodies: HRP-linked anti-rabbit IgG (7074, Cell Signaling Technology) and HRP-linked anti-mouse IgG (Jackson ImmunoResearch Laboratories, West Grove, PA, USA).

### 2.6. Mouse Studies

APPswe/PS1dE9 (APP/PS1) double transgenic mice were purchased from Jackson Laboratory (No. 005864, Bar Harbor, ME, USA). Breeding was conducted using female transgenic mice and their male wild-type siblings. Mice were maintained under a 12 h/12 h light/dark cycle in constant conditions of temperature (24 °C) and humidity (55–65%) with free access to food and water. All procedures were approved by the Institutional Animal Care and Use Committee at the National Research Institute of Chinese Medicine (IACUC No.:105-417-1).

### 2.7. Feeding Protocol

HH-F3 powder was dissolved in H_2_O. HH-F3 (300 mg/kg/day) or H_2_O were administered orally to wild-type and APP/PS1 mice for 30 days.

### 2.8. Thioflavin-S (ThS) Fluorescent Staining in Brain Sections

Dry sections of mouse brains were incubated with fresh and filtered 1% ThS solution for 60 min, followed by washing twice with 70% ethanol and twice with water.

### 2.9. Detection of Soluble and Insoluble Aβ

Frozen cerebral hemispheres were homogenized in homogenization buffer (320 mM sucrose, 2 mM EDTA, 20 mM Tris-HCl, pH 7.4) with protease and phosphatase inhibitors (Roche, Basel, Switzerland). SDS-soluble (2% SDS) fractions and FA-extracted (70% formic acid) fractions were obtained by stepwise ultracentrifugations.

### 2.10. L1000 Expression Profiling

The gene expression of the HT29 cells treated with 5 µg/mL HH-F3 for 6 h was profiled using the L1000 platforms by Genometry Inc., Cambridge, MA, USA [24]. In short, mRNA transcripts of HH-F3 treated cells were captured from the whole cell lysates by o ligo-dT plates. The cDNAs were generated by reverse transcription from mRNA and then amplified using PCR. The PCR amplicon was then hybridized to barcoded Luminex beads to exhibit the expression levels of specific genes. The expression of 978 landmark genes was analyzed.

### 2.11. Gene Set Enrichment Analysis (GSEA)

GSEA analysis was performed in GSEA software version 4.0.3 (Broad Institute, MA, USA). The study was proceeded using the C2 gene set collections from the MSigDB v.7.2. with 1000 permutations.

### 2.12. C. elegans Strains

All strains were maintained at 20 °C on NGM plates seeded with *E. coli* OP50. NGM plates with UV-killed bacteria were used for all experiments with HH-F3. The following strains were used in the study: Wild-type Bristol N2; DA2123: *adIs*2122[*gfp::lgg*-1 + *rol-*6]; OP433: *wgIs*[*hlh-*30*::TY*1*::EGFP::*3*xFLAG* + *unc*-119(+)]; CF1038: *daf*-*16(mu86)*, and AM140: *rmIs*132[*unc-*54*p::Q*35*::YFP*]. DA2123, OP433, and wild-type *Caenorhabditis elegans* (N2) strains were obtained from the Caenorhabditis Genetic Center at the University of Minnesota.

### 2.13. GFP::LGG-1 Puncta Quantification

GFP::LGG-1 foci formation was visualized in L3 stage animals (DA2123) that were treated with either control (vehicle) or HH-F3 from hatching. Fluorescence images of the animals were taken via an Olympus BX63 microscope (Olympus, Tokyo, Japan) using Microsuite software (Olympus). Fifteen to twenty animals were scored for seam cell GFP::LGG-1 puncta accumulation.

### 2.14. HLH-30::GFP Nuclear-Cytoplasmic Ratio (N/C Ratio) Quantification

Synchronized worms carrying integrated *hlh-30::gfp* arrays were treated either in vehicle or HH-F3 from hatching. Fluorescence images of the Day 1 adult animals were taken and scored blindly for the nuclear accumulation of HLH-30::GFP protein in the intestinal cells. The quantification was perforemd by measuring the total GFP fluorescence intensity of the entire cell and the nucleus area of the first six intestinal cells (Int1* and Int2* cells) using Olympus Microsuite software. Cytosolic GFP intensity was calculated by the following equation, $\frac{\mathrm{IntDen}\;\left(\mathrm{whole}\;\mathrm{cell}\right)-\mathrm{IntDen}\;\left(\mathrm{nucleus}\right)}{\mathrm{Area}\;\left(\;\mathrm{whole}\;\mathrm{cell}\right)-\mathrm{Area}\;\left(\mathrm{nucleus}\right)}$. The N/C ratio of HLH-30 in a given cell was obtained by dividing the nuclear signal by the cytosolic signal. At least 50 animals (1–4 cells per worm) were scored per experiment.

### 2.15. Mobility Analysis in C. elegans

Thrashing assays were carried on at least 12 worms. Individual stage-synchronized worms were placed in M9 buffer. Thrashes produced by each worm for one minute were counted after one minute equilibration period. A single thrash was defined as the bending of the body to the outermost angle and then back to the initial posture. Experimental data are shown as mean ± SEM. Statistical comparisons were conducted using Student’s *t*-test.

### 2.16. Lifespan Analysis in C. elegans

Lifespan analyses were conducted at 20 °C as described previously [25,26], and 60–90 animals were tested in each experiment. The viability of the worms was scored every two days. In all experiments, the pre-fertile period of adulthood was used as day 0 for lifespan analysis. Stata 12 (StataCorp, College Station, Texas, USA) software was used for statistical analysis to determine the means and percentiles. In all cases, *p* values were calculated using the log-rank (Mantel–Cox) method.

## 3. Results

### 3.1. GP Extract Inhibits the Secretion of Amyloid-Aβs in the Human Neuroblastoma SH-SY5Y-APP695 Cells

**Figure 1:**
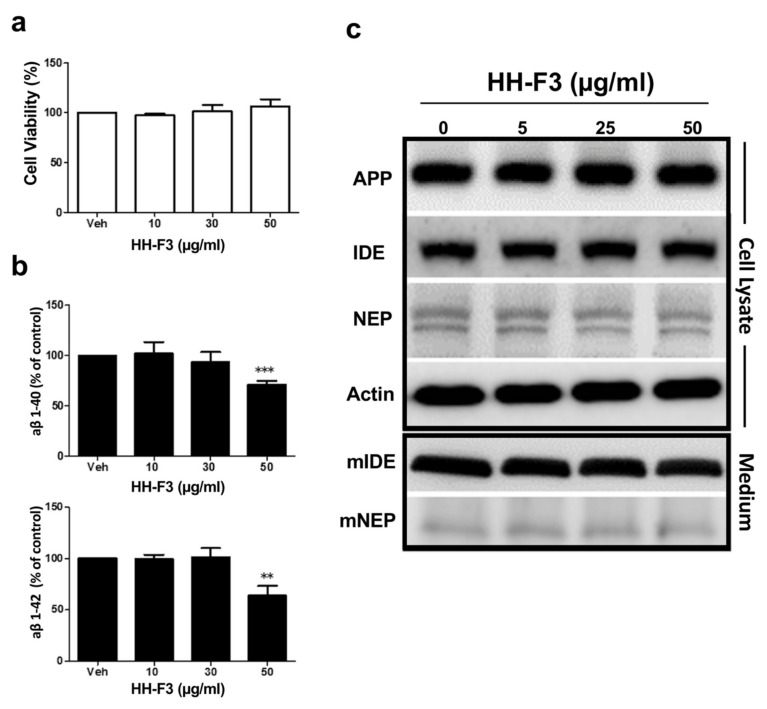
GP extract HH-F3 reduced Aβ40 and Aβ42 secretion from SH-SY5Y-APP_695_ cells. SH-SY5Y-APP_695_ cells were treated with varying HH-F3 concentrations at 0, 10, 30, and 50 μg/mL for 24 h. (**a**) Effects of HH-F3 on cell viability. (**b**) Levels of Aβ1–40 and Aβ1–42 were assayed by ELISA in media of SH-SY5Y-APP_695_ cells treated with HH-F3. (**c**) NEP and IDE were measured by Western blot in the cell lysates and conditioned medium of SH-SY5Y-APP_695_ cells treated with indicated concentrations of HH-F3. Data were analyzed by Student’s *t*-test. Levels of significance are shown as ** *p*<0.01 and *** *p*<0.001.

Recently, the study by Wu et al. demonstrated that the GP extract, HH-F3, significantly reduces Aβ secretion in AD patient iPSC-derived neurons [22]. Previous studies have shown that the overexpression of APP_695_ in human SH-SY5Y cells significantly increases Aβ40 and Aβ42 secretion [27]. To further explore the molecular mechanisms of HH-F3 to reduce the AD-associated phenotypes, we used SH-SY5Y cells that stably expressed wild-type human APP_695_ as the cell culture model. We first treated SH-SY5Y-APP_695_ cells with HH-F3 at concentrations of 10, 30, and 50 µg/mL for 24 h. The cytotoxic effect of HH-F3 was evaluated by cell viability assay. No cytotoxic effect was observed in SH-SY5Y-APP_695_ cells even at the highest concentration of HH-F3 (Figure 1a). We then assessed the impact of HH-F3 on the secretion of Aβ40 and Aβ42 in SH-SY5Y-APP_695_ cultured medium by ELISA assay. Our results indicated that HH-F3 treatment for 24 h markedly reduced Aβ40 and Aβ42 secretion at the dosage of 50 µg/mL (Figure 1b), which is also the same effective concentration applied to the AD-iPSC derived neurons [22]. Thus, SH-SY5Y-APP_695_ cells serve as a proper cell model to further study the molecular mechanisms of HH-F3 in the regulation of amyloid secretion. We next examined whether full-length APP levels in SH-SY5Y-APP_695_ cells were affected by HH-F3 treatment. As shown in Figure 1c, the amounts of full-length APP were not changed by the treatment of HH-F3. Meanwhile, HH-F3 did not affect the levels of major amyloid degrading proteases, such as insulin-degrading enzyme (IDE) and neprilysin (NEP), neither in the cell lysate of SH-SY5Y-APP_695_ cells nor in the conditioned medium (Figure 1c).

### 3.2. GP Extract Reduces the Plaque Formation in the Cerebral Cortex of APP/PS1 Transgenic Mice

**Figure 2:**
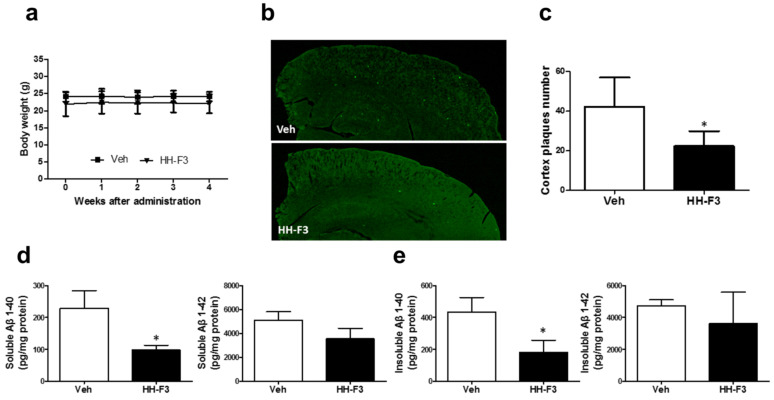
GP extract HH-F3 diminished amyloid plaque formation and Aβ levels in the cerebral cortex of APP/PS mice. HH-F3 at 0 or 300 mg kg^− 1^ per day was given to 5-month-old APP/PS1 for 30 days. (**a**) The body weights of APP/PS1 mice fed with or without HH-F3. (**b**) ThS-stained Aβ plaques in the cerebral cortex region of APP/PS1 mice fed with or without HH-F3. (**c**) The number of relative total areas in the cerebral cortex region. (**d**) Levels of Aβ40 and Aβ42 in the soluble fraction from the brain homogenates of APP/PS1 mice treated with or without HH-F3. (**e**) Levels of Aβ40 and Aβ42 in the insoluble fraction from the brain homogenates of APP/PS1 mice treated with or without HH-F3. Data were analyzed by Student’s *t*-test. Levels of significance are shown as * *p* < 0.05.

We then investigated the effects of HH-F3 in APP/PS mice model of AD. The APP/PS1 transgenic mouse is widely used in various aspects of AD-related study. In this study, HH-F3 was administrated to 140-day-old APP/PS1 mice orally at 300 mg/kg/day for 30 days. There were no significant differences in the body weight between the control and HH-F3-treated groups after a 30-day treatment (Figure 2a). To test whether HH-F3 could reduce the Aβ deposition in APP/PS1 mice, we performed thioflavin-S (ThS) fluorescent staining to detect Aβ plagues in the cerebral hemisphere of APP/PS1 mice fed with or without HH-F3. Our results indicated that Aβ deposit formation in the cerebral hemisphere is markedly reduced by 48% after a 30-day HH-F3 treatment (Figure 2b,c). Furthermore, through Aβ1-40 and Aβ1-42 ELISA assays, we found that both soluble and insoluble Aβ1-40 levels in the cerebral cortex were significantly reduced in the HH-F3-treated group (Figure 2d,e). The amounts of soluble Aβ1-42 slightly decreased in the HH-F3-treated mice. However, the data were not statistically significant (Figure 2d).

### 3.3. GP Extract Activates AMPK in U87 Cells and the Cerebral Cortex of APP/PS1 Mice

To gain an overview of the altered pathways after HH-F3 treatment, differentially expressed genes of HT29 cells treated with 50 µg/mL HH-F3 were subjected to Gene Set Enrichments Analysis (GSEA). The results indicated that genes associated with HD, AD, and AMPK signaling were significantly enriched in HH-F3-treated cells (Supplementary Figure S1), suggesting that HH-F3 might reduce neurodegeneration via the activation of AMPK pathways.

**Figure 3:**
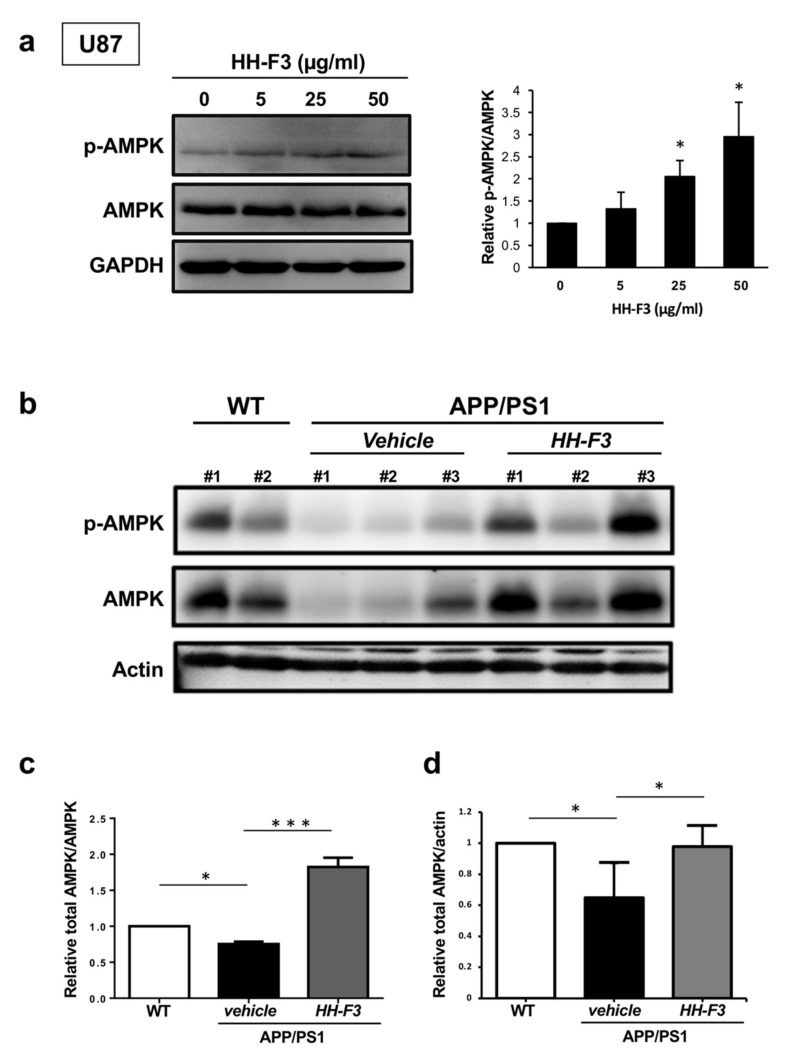
GP extract HH-F3 enhanced AMPK activity in U87 cells and APP/PS mice. (**a**) Western blot analysis of AMPK phosphorylation (Thr172) and AMPK in U87 cells treated with 0, 5, 25, and 50 μg/mL of HH-F3 for 24 h. Mean ± SD for three biological replicates. Data were analyzed by Student’s *t*-test. * *p* < 0.05. (**b**) Western blot analysis of AMPK phosphorylation (Thr172) and AMPK in the cerebral cortex of APP/PS1 mice treated with or without HH-F3 for 30 days. Western blot quantification of AMPK phosphorylation (Thr172) (**c**) and AMPK (**d**) in the cerebral cortex of wild-type mice and APP/PS1 mice treated with or without HH-F3. The results are expressed as mean ± SD. * *p* < 0.05 and *** *p* < 0.001. The mean value of pAMPK/AMPK ratios from wild-type mice was normalized to one.

As described earlier, the GSEA analysis of HH-F3-treated cells revealed significantly enriched AMPK pathways. To further confirm the effects of HH-F3 in AMPK signaling, glial U87 cells were treated with HH-F3 at a concentration of 10, 25, and 50 µg/mL for 24 h. We accessed the activity of AMPK by measuring the phosphorylation at the Thr172 of AMPK (pAMPK). HH-F3 treatment significantly activated AMPK in U87 cells, as shown by the elevated pAMPK/AMPK ratios (Figure 3a). Previous studies have indicated that AMPK signaling pathways [28] are dysregulated in the brains of APP/PS1 mouse model and human AD patients. Thus, we examine if HH-F3 reduces AD pathology by elevating AMPK signaling in APP/PS1 mice. Our results demonstrated that the levels of phosphorylated AMPK and total AMPK in the cerebral cortex of APP/PS1 mice were reduced (Figure 3b), suggesting the downregulation of AMPK signaling pathway. Intriguingly, a 30-day HH-F3 treatment could markedly restore the levels of both pAMPK and AMPK (Figure 3b), supporting that HH-F3 might act as an AMPK activator to reduce pathological conditions in APP/PS1 mice.

### 3.4. Autophagy Is Elevated by GP Extract Both in Cells and C. elegans

**Figure 4:**
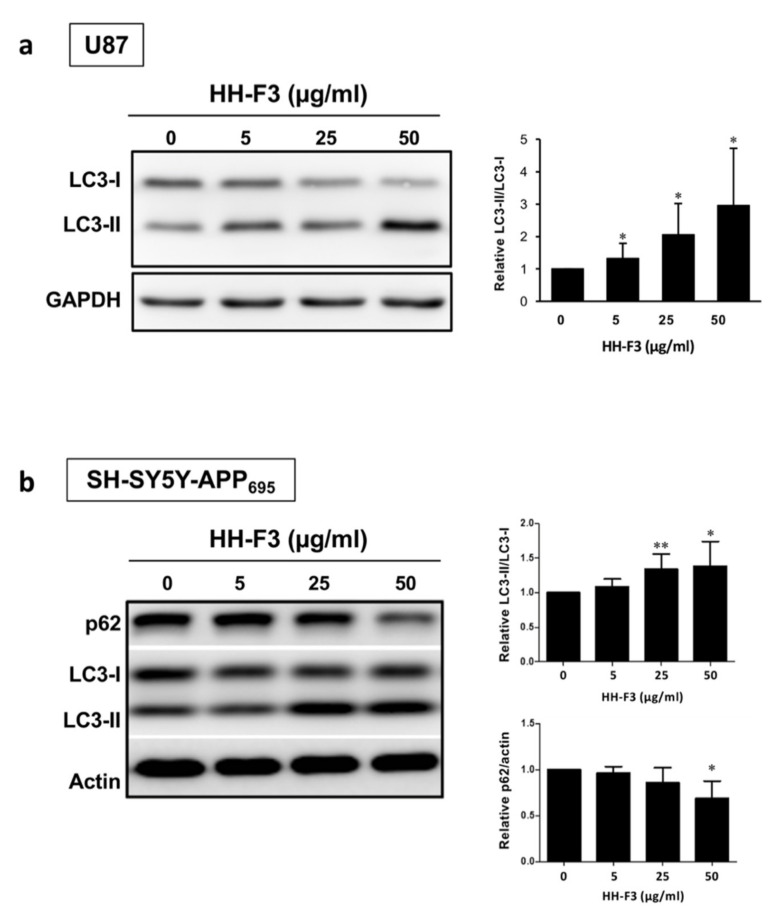
GP extract HH-F3 activated autophagy in U87 and SH-SY5Y-APP_695_ cells. (**a**) The expression of LC3-I and LC3-II in U87 cells treated with 0, 5, 25, and 50 μg/mL for 24 h. The bands were analyzed by quantitative densitometry. The ratio of LC3-II/LC3-I was determined after normalization to GAPDH. (**b**) Western blot analysis of LC3-I, LC3-II, and p62 in SH-SY5Y-APP_695_ cells treated with 0, 5, 25, and 50 μg/mL for 24 h. The folds of the mean grayscale of LC3-II/LC3-I and p62 to actin among treatments are shown on the right. Mean ± SD for 2–3 biological replicates. * *p* < 0.05, ** *p* < 0.01, Student’s *t*-test.

Several pieces of evidence have indicated that autophagy dysregulation occurs in both AD patients and animal models [29,30,31]. Moreover, numerous studies have reported that the genetic or pharmacological activations of autophagy could reduce amyloid accumulation and prevent cognitive decline in AD-mouse models [32,33,34,35]. According to our data shown above, HH-F3 treatment could increase the activity of AMPK, one of the key autophagy regulators. Thus, we tested whether HH-F3 ameliorates AD pathology through activation of autophagy. Neurons and glia are the two major types of cells in the brain. Glial cells, such as astrocytes, microglia, and oligodendrocytes, are also critical in AD pathogenesis [36]. Research has shown that autophagy in glial cells plays a key role in reducing extracellular Aβ around neurons [37,38]. Here, we monitored the autophagic activity in the HH-F3-treated glial U87 cells and neuronal SH-SY5Y-APP_695_ cells by analyzing the turnover of microtubule-associated protein 1A/1B-light chain 3 (LC3), a maker of autophagosomal membrane. As shown in Figure 4, an HH-F3-dependent increase in the levels of LC3-II, the lipidated LC3, suggests that the induction of autophagy was enhanced by HH-F3 treatment in both U87 and SH-SY5Y-APP_695_ cells. Furthermore, we found that p62 levels were significantly reduced in SH-SY5Y-APP_695_ cells at the dosage of 50 µg/mL HH-F3 (Figure 4b), indicating that HH-F3 could indeed activate autophagy flux.

**Figure 5:**
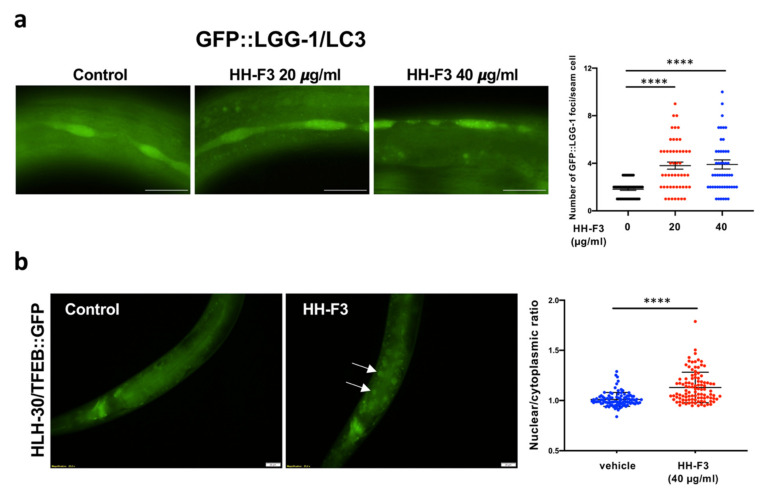
GP extract HH-F3 activated autophagy in *C. elegans.* (**a**) GFP::LGG-1/LC3 punctae were examined in the hypodermal seam cells of L3 larval stage animals expressing *lgg-*1*p::gfp::lgg-*1 treated with 0, 20, and 40 μg/mL HH-F3 from hatching (n > 20). (**** *p* < 0.0001, One-way ANOVA test) (**b**) Distribution of HLH-30/TFEB::GFP was visualized in Day 1 adult worms expressing *hlh-*30*::gfp* treated with 40 μg/mL HH-F3 from hatching. The nuclear/cytosol ratios of HLH-30::GFP in the intestinal cells are shown. (**** *p* < 0.0001, Chi-squared test).

Next, we examined whether HH-F3 could activate autophagy activity through an evolutionarily conserved mechanism. To do so, we performed the HH-F3 treatments in the nematode *C. elegans*. We used the transgenic worms carrying GFP::LGG-1, the worm homolog of LC3, to detect autophagy activity. Increased levels of GFP::LGG-1 puncta commonly represent the activation of autophagy. Here, GFP::LGG-1 mutants were treated with 0, 20, and 40 µg/mL HH-F3 from hatching. After a two-day HH-F3 treatment, the levels of LGG-1/LC3 puncta in the seam cells were remarkably increased (Figure 5a), indicating autophagy activation.

We further tested whether HLH-30/TFEB, the master transcription factor for autophagy and lysosome biogenesis, is involved in HH-F3-induced autophagy activation. The nuclear localization of HLH-30/TFEB, which moves into the nucleus upon stimuli, was analyzed by using transgenic animals expressing HLH-30::GFP protein. Our results indicated that HH-F3 could markedly trigger HLH-30/TFEB nuclear translocation in *C. elegans* (Figure 5b), suggesting that HLH-30/TFEB might mediate the HH-F3-induced autophagy.

### 3.5. GP Extract Extended Lifespan in C. elegans in a Daf-16-Independent Manner

**Figure 6:**
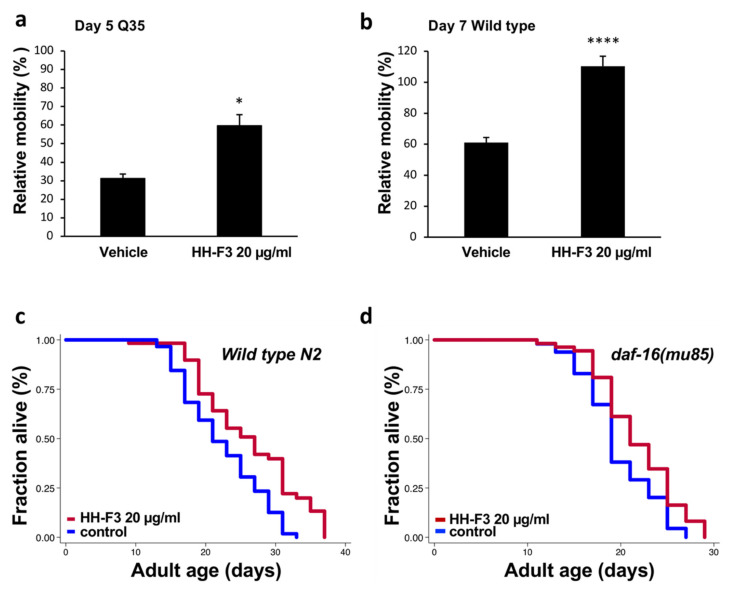
GP extract HH-F3 increased the health span and lifespan of *C. elegans* through a daf-16/FOXO-independent pathway. (**a**) Relative mobility of Day 5 Q35 animals treated with vehicle control or 20 μg/mL HH-F3. Data are mean ± SEM as a percentage of the Day 1 vehicle control. (**b**) Relative mobility of Day 7 wild-type animals treated with vehicle control or 20 μg/mL HH-F3. Data are mean ± SEM as a percentage of the Day 1 vehicle control. (* *p* < 0.05, **** *p* < 0.0001, Student’s *t*-test) (**c**) Lifespan analysis of wild-type N2 worms treated with or without 20 μg/mL HH-F3. (**d**) Lifespan analysis of *daf-16* mutants treated with or without 20 μg/mL HH-F3. The detailed results of lifespan analysis are shown in Supplementary Table S1.

As the above results show, HH-F3 could promote autophagy activity across species. The stimulation of autophagy has been shown to enhance the turnover of aggregated proteins, such as TDP-43 and huntingtin. Therefore, we asked whether HH-F3 could reduce the pathological phenotypes induced by disease-associated protein aggregation in other model organisms. Here, we used *C. elegans* expressing fluorescently tagged polyglutamine (polyQ) in the body-muscle cells to study the effects of HH-F3 in polyQ pathogenesis. The transgenic animals carrying 35 polyglutamine repeats (Q35) were treated with 20 µg/mL HH-F3 from L4 larval stage, and the mobility of Day 5 adults was determined by thrashing assay. In the Q35 mutants treated with vehicle, the locomotion decreased to 30% in Day 5 Q35 worms. However, Day 5 Q35 worms treated HH-F3 still maintain 70% of mobility in Day 1 adult animals (Figure 6a). Thus, HH-F3 could significantly lessen the mobility decline caused by polyQ-mediated toxicity in the muscle cells.

Increasing evidence has indicated that autophagy might serve as a common downstream effector in aging processes. Since HH-F3 could activate autophagy in the mammalian cells and nematodes, we tested whether HH-F3 could slow down the aging process and extend lifespan in *C. elegans*. First, we verified if HH-F3 treatment could prevent mobility decline during aging. We performed thrashing assays on Day 1 and Day 7 adult wild-type N2 worms treated with vehicle or 20 µg/mL HH-F3. As shown in Figure 6b, the mobility in Day 7 N2 worms fed with the vehicle was reduced by 40% compared with the Day 1 animals. However, there was no significant difference between the mobility of Day 1 and Day 7 worms treated with HH-F3 (Figure 6b), indicating that HH-F3 treatment could prevent mobility decline in aged animals. Next, we performed lifespan analysis on wild-type N2 animals treated with 20 µg/mL HH-F3. We found that 20 µg/mL HH-F3 significantly increases animals’ lifespan by 14–16% (Figure 6c, Table S1).

DAF-16, a FOXO transcription factor in *C. elegans*, is the key mediator for several longevity pathways, such as insulin/IGF-1 signaling and germline signaling. We then further investigated whether DAF-16/FOXO is required in HH-F3-induced lifespan extension. We thus performed lifespan analysis in *daf-*16 null mutants treated with vehicle or 20 µg/mL HH-F3. As shown in Figure 6d, HH-F3 could still increase the lifespan of *daf-*16 mutants by 11.3%, suggesting that the lifespan extension induced by HH-F3 treatment was not dependent on *daf-16/FOXO*.

## 4. Discussion

Our study has shown that GP extract, HH-F3, markedly reduced amyloid-β secretion in both SH-SY5Y-APP_695_ cells and APP/PS1 mice. Furthermore, amyloid plaque formation in APP/PS1 mice was lessened after a 30-day HH-F3 treatment, suggesting that HH-F3 is a potential therapeutic candidate for AD treatment. To elucidate the molecular mechanisms of HH-F3 to reduce AD pathology, we identified that HH-F3 could activate autophagy in U87 and SH-SY5Y-APP_695_ cells. Moreover, the activation of autophagy by HH-F3 was observed not only in the mammalian cells but also in *C. elegans*. Our findings have suggested that HH-F3 might promote autophagy through a conserved pathway across species, further supporting its plausible application in humans.

Loss of protein homeostasis (proteostasis) has been described as one of the hallmarks of aging [2,39]. Since the autophagy-lysosomal pathway is one of the main cellular mechanisms in maintaining proteostasis [40,41], autophagy activation has been thought to be beneficial to longevity [16]. Indeed, studies from various model organisms have shown the essential role of autophagy in the regulation of longevity [15,16]. Furthermore, enhancing autophagic activity by overexpressing autophagy genes could extend the lifespan of flies and mice [17,42]. Since AMPK and TFEB/HLH-30 are two critical regulators in autophagic activity [18], presumably, AMPK and TFEB/HLH-30 might also affect longevity regulation. Indeed, several lines of evidence have also demonstrated that AMPK and HLH-30/TFEB are both involved in lifespan regulation in *C. elegans* [43,44,45]. Moreover, the overexpression of AMPK and HLH-30/TFEB could extend lifespan in *C. elegans* [43,44,46]. Thus, pharmacological activation of AMPK or HLH-30/TFEB might also promote lifespan and health span in animals. Given the fact that HH-F3 could activate AMPK and HLH-30/TFEB, we presumed that HH-F3 might have longevity effects. Indeed, our results have indicated that HH-F3 significantly delayed the mobility decline and extended the lifespan of wild-type animals. Furthermore, HH-F3 greatly reduced polyQ pathology in *C. elegans*, supporting the negative effect of HH-F3 in age-associated decline of proteostasis. Through a genetic epistasis analysis, we further found that *daf-16/FOXO* transcription factor is not required in the longevity effect of HH-F3. Our results in both AD models and *C. elegans* suggest that the GP extract HH-F3 might act as an autophagy activator to maintain proteostasis, slowing down the aging process and delaying age-related disease onset. Therefore, HH-F3 may be a potential pharmacological candidate for the future development of anti-aging drugs.

## Data Availability

The lifespan data present in this study are available in Table S1. All data generated or analyzed during the current study are available from the corresponding author upon reasonable request.

## Supplementary Materials

The following are available online at https://www.mdpi.com/article/10.3390/nu13124317/s1, Table S1: Statistical data for *C. elegans* lifespan experiments. Figure S1: Gene set enrichment analysis (GSEA) of the microarray data from HT29 cells treated with HH-F3.

## References

[1] Khan S.S., Singer B.D., Vaughan D.E. (2017). Molecular and physiological manifestations and measurement of aging in humans. Aging Cell.

[2] Lopez-Otin C., Blasco M.A., Partridge L., Serrano M., Kroemer G. (2013). The hallmarks of aging. Cell.

[3] Serrano-Pozo A., Frosch M.P., Masliah E., Hyman B.T. (2011). Neuropathological alterations in Alzheimer disease. Cold Spring Harb. Perspect. Med..

[4] Glick D., Barth S., Macleod K.F. (2010). Autophagy: Cellular and molecular mechanisms. J. Pathol..

[5] Halama A., Kulinski M., Dib S.S., Zaghlool S.B., Siveen K.S., Iskandarani A., Zierer J., Prabhu K.S., Satheesh N.J., Bhagwat A.M. (2018). Accelerated lipid catabolism and autophagy are cancer survival mechanisms under inhibited glutaminolysis. Cancer Lett..

[6] Caballero B., Wang Y., Diaz A., Tasset I., Juste Y.R., Stiller B., Mandelkow E.M., Mandelkow E., Cuervo A.M. (2018). Interplay of pathogenic forms of human tau with different autophagic pathways. Aging Cell.

[7] Deng Z., Sheehan P., Chen S., Yue Z. (2017). Is amyotrophic lateral sclerosis/frontotemporal dementia an autophagy disease?. Mol. Neurodegener..

[8] Menzies F.M., Fleming A., Rubinsztein D.C. (2015). Compromised autophagy and neurodegenerative diseases. Nat. Rev. Neurosci..

[9] Nixon R.A., Yang D.S. (2011). Autophagy failure in Alzheimer’s disease--locating the primary defect. Neurobiol. Dis..

[10] Schaeffer V., Lavenir I., Ozcelik S., Tolnay M., Winkler D.T., Goedert M. (2012). Stimulation of autophagy reduces neurodegeneration in a mouse model of human tauopathy. Brain.

[11] Lin A.L., Jahrling J.B., Zhang W., DeRosa N., Bakshi V., Romero P., Galvan V., Richardson A. (2017). Rapamycin rescues vascular, metabolic and learning deficits in apolipoprotein E4 transgenic mice with pre-symptomatic Alzheimer’s disease. J. Cereb. Blood Flow Metab..

[12] Congdon E.E., Wu J.W., Myeku N., Figueroa Y.H., Herman M., Marinec P.S., Gestwicki J.E., Dickey C.A., Yu W.H., Duff K.E. (2012). Methylthioninium chloride (methylene blue) induces autophagy and attenuates tauopathy in vitro and in vivo. Autophagy.

[13] Boland B., Kumar A., Lee S., Platt F.M., Wegiel J., Yu W.H., Nixon R.A. (2008). Autophagy induction and autophagosome clearance in neurons: Relationship to autophagic pathology in Alzheimer’s disease. J. Neurosci..

[14] Martin D.D., Ladha S., Ehrnhoefer D.E., Hayden M.R. (2015). Autophagy in Huntington disease and huntingtin in autophagy. Trends Neurosci..

[15] Nakamura S., Yoshimori T. (2018). Autophagy and Longevity. Mol. Cells.

[16] Rubinsztein D.C., Marino G., Kroemer G. (2011). Autophagy and aging. Cell.

[17] Pyo J.O., Yoo S.M., Ahn H.H., Nah J., Hong S.H., Kam T.I., Jung S., Jung Y.K. (2013). Overexpression of Atg5 in mice activates autophagy and extends lifespan. Nat. Commun..

[18] Fernandez A.F., Sebti S., Wei Y., Zou Z., Shi M., McMillan K.L., He C., Ting T., Liu Y., Chiang W.C. (2018). Disruption of the beclin 1-BCL2 autophagy regulatory complex promotes longevity in mice. Nature.

[19] Hsu W.H., Liao S.C., Chyan Y.J., Huang K.W., Hsu S.L., Chen Y.C., Siu M.L., Chang C.C., Chung Y.S., Huang C.F. (2019). Graptopetalum paraguayense Inhibits Liver Fibrosis by Blocking TGF-beta Signaling In Vivo and In Vitro. Int. J. Mol. Sci..

[20] Duh P.D., Lin S.L., Wu S.C. (2011). Hepatoprotection of Graptopetalum paraguayense E. Walther on CCl(4)-induced liver damage and inflammation. J. Ethnopharmacol..

[21] Su L.J., Chang C.C., Yang C.H., Hsieh S.J., Wu Y.C., Lai J.M., Tseng T.L., Huang C.Y., Hsu S.L. (2013). Graptopetalum paraguayense ameliorates chemical-induced rat hepatic fibrosis in vivo and inactivates stellate cells and Kupffer cells in vitro. PLoS ONE.

[22] Wu P.C., Fann M.J., Tran T.T., Chen S.C., Devina T., Cheng I.H., Lien C.C., Kao L.S., Wang S.J., Fuh J.L. (2019). Assessing the therapeutic potential of Graptopetalum paraguayense on Alzheimer’s disease using patient iPSC-derived neurons. Sci. Rep..

[23] Hsu W.H., Chang C.C., Huang K.W., Chen Y.C., Hsu S.L., Wu L.C., Tsou A.P., Lai J.M., Huang C.Y. (2015). Evaluation of the medicinal herb Graptopetalum paraguayense as a treatment for liver cancer. PLoS ONE.

[24] Subramanian A., Narayan R., Corsello S.M., Peck D.D., Natoli T.E., Lu X., Gould J., Davis J.F., Tubelli A.A., Asiedu J.K. (2017). A Next Generation Connectivity Map: L1000 Platform and the First 1,000,000 Profiles. Cell.

[25] Hsu A.L., Murphy C.T., Kenyon C. (2003). Regulation of aging and age-related disease by DAF-16 and heat-shock factor. Science.

[26] Apfeld J., Kenyon C. (1999). Regulation of lifespan by sensory perception in Caenorhabditis elegans. Nature.

[27] Lopez Sanchez M.I.G., Waugh H.S., Tsatsanis A., Wong B.X., Crowston J.G., Duce J.A., Trounce I.A. (2017). Amyloid precursor protein drives down-regulation of mitochondrial oxidative phosphorylation independent of amyloid beta. Sci. Rep..

[28] Ma T., Chen Y., Vingtdeux V., Zhao H., Viollet B., Marambaud P., Klann E. (2014). Inhibition of AMP-activated protein kinase signaling alleviates impairments in hippocampal synaptic plasticity induced by amyloid beta. J. Neurosci..

[29] Lee S., Sato Y., Nixon R.A. (2011). Primary lysosomal dysfunction causes cargo-specific deficits of axonal transport leading to Alzheimer-like neuritic dystrophy. Autophagy.

[30] Ma J.F., Huang Y., Chen S.D., Halliday G. (2010). Immunohistochemical evidence for macroautophagy in neurones and endothelial cells in Alzheimer’s disease. Neuropathol. Appl. Neurobiol..

[31] Shin J.Y., Park H.J., Kim H.N., Oh S.H., Bae J.S., Ha H.J., Lee P.H. (2014). Mesenchymal stem cells enhance autophagy and increase beta-amyloid clearance in Alzheimer disease models. Autophagy.

[32] Rocchi A., Yamamoto S., Ting T., Fan Y., Sadleir K., Wang Y., Zhang W., Huang S., Levine B., Vassar R. (2017). A Becn1 mutation mediates hyperactive autophagic sequestration of amyloid oligomers and improved cognition in Alzheimer’s disease. PLoS Genet..

[33] Caccamo A., De Pinto V., Messina A., Branca C., Oddo S. (2014). Genetic reduction of mammalian target of rapamycin ameliorates Alzheimer’s disease-like cognitive and pathological deficits by restoring hippocampal gene expression signature. J. Neurosci..

[34] Caccamo A., Majumder S., Richardson A., Strong R., Oddo S. (2010). Molecular interplay between mammalian target of rapamycin (mTOR), amyloid-beta, and Tau: Effects on cognitive impairments. J. Biol. Chem..

[35] Majumder S., Richardson A., Strong R., Oddo S. (2011). Inducing autophagy by rapamycin before, but not after, the formation of plaques and tangles ameliorates cognitive deficits. PLoS ONE.

[36] Dzamba D., Harantova L., Butenko O., Anderova M. (2016). Glial Cells—The Key Elements of Alzheimer s Disease. Curr. Alzheimer Res..

[37] Simonovitch S., Schmukler E., Bespalko A., Iram T., Frenkel D., Holtzman D.M., Masliah E., Michaelson D.M., Pinkas-Kramarski R. (2016). Impaired Autophagy in APOE4 Astrocytes. J. Alzheimers Dis..

[38] Xiao Q., Yan P., Ma X., Liu H., Perez R., Zhu A., Gonzales E., Burchett J.M., Schuler D.R., Cirrito J.R. (2014). Enhancing astrocytic lysosome biogenesis facilitates Abeta clearance and attenuates amyloid plaque pathogenesis. J. Neurosci..

[39] Hipp M.S., Kasturi P., Hartl F.U. (2019). The proteostasis network and its decline in ageing. Nat. Rev. Mol. Cell Biol..

[40] Dikic I. (2017). Proteasomal and Autophagic Degradation Systems. Annu. Rev. Biochem..

[41] Kaushik S., Cuervo A.M. (2015). Proteostasis and aging. Nat. Med..

[42] Simonsen A., Cumming R.C., Brech A., Isakson P., Schubert D.R., Finley K.D. (2008). Promoting basal levels of autophagy in the nervous system enhances longevity and oxidant resistance in adult Drosophila. Autophagy.

[43] Apfeld J., O’Connor G., McDonagh T., DiStefano P.S., Curtis R. (2004). The AMP-activated protein kinase AAK-2 links energy levels and insulin-like signals to lifespan in C. elegans. Genes Dev..

[44] Lapierre L.R., De Magalhaes Filho C.D., McQuary P.R., Chu C.C., Visvikis O., Chang J.T., Gelino S., Ong B., Davis A.E., Irazoqui J.E. (2013). The TFEB orthologue HLH-30 regulates autophagy and modulates longevity in Caenorhabditis elegans. Nat. Commun..

[45] Weir H.J., Yao P., Huynh F.K., Escoubas C.C., Goncalves R.L., Burkewitz K., Laboy R., Hirschey M.D., Mair W.B. (2017). Dietary Restriction and AMPK Increase Lifespan via Mitochondrial Network and Peroxisome Remodeling. Cell Metab..

[46] Kuo C.T., You G.T., Jian Y.J., Chen T.S., Siao Y.C., Hsu A.L., Ching T.T. (2020). AMPK-mediated formation of stress granules is required for dietary restriction-induced longevity in Caenorhabditis elegans. Aging Cell.

