# Supplementary material for: Graptopetalum paraguayense Extract Ameliorates Proteotoxicity in Aging and Age-Related Diseases in Model Systems
Source: Nutrients. 2021 Nov 29;13(12):4317. doi: 10.3390/nu13124317 (PMC8706064; doi:10.3390/nu13124317)
Supplement: Supplementary file 1 [file nutrients-13-04317-s001.zip › nutrients-1441327-supplementary.pdf]

Supplementary Table S1 |
Statistical data for *C. elegans* lifespan experiments

strain name	treatment	mean	p (Log-rank test)	%change	75%	n
N2	0	20.1 ± 0.51			23	60/72
N2	*2*	22.4 ± 0.78	0.0159	11.4	25	54/72
N2	*20*	23.3 ± 1.21	0.0038	15.9	27	30/72
N2	0	24.3 ± 0.59			29	57/72
N2	*2*	26.2 ± 0.33	0.0384	7.8	29	57/72
N2	*20*	26.5 ± 0.46	0.0028	9.1	29	60/96
N2	*0*	25.7 ± 0.84			29	45/72
N2	*20*	29.4 ± 0.77	0.0001	14.4	31	42/72
N2	*0*	22.1 ± 1.49			27	57/72
N2	*20*	25.6 ± 2.01	0.0009	15.8	31	48/72
daf-16(mu86)	*0*	19.5 ± 0.50			23	46/72
daf-16(mu86)	*20*	21.7 ± 1.06	0.0081	11.3	25	50/72

Supplementary Figure S1

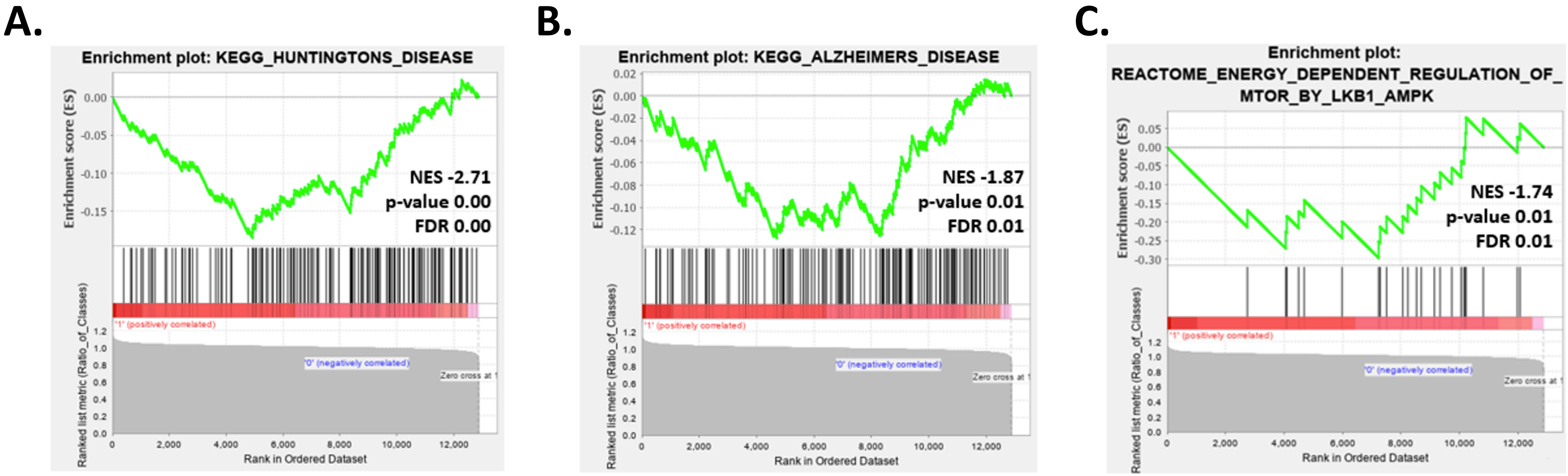

Figure S1. Gene set enrichment analysis (GSEA) of the microarray data from HT29 cells treated with HH-F3. GSEA enrichment plots of disease-related gene sets include **(A)** Huntington's disease (KEGG: 05016), **(B)** Alzheimer's disease (KEGG: 05010), and **(C)** energy dependent regulation of mTOR by LKB1-AMPK. ES: Enrichment scores; NES: Normalized enrichment scores; FDR: False discovery rate.
